# Significance of Initial Chest CT Severity Score (CTSS) and Patient Characteristics in Predicting Outcomes in Hospitalized COVID-19 Patients: A Single Center Study

**DOI:** 10.3390/v16111683

**Published:** 2024-10-29

**Authors:** Aleksandra Milenkovic, Simon Nikolic, Zlatan Elek, Jelena Aritonovic Pribakovic, Aleksandra Ilic, Kristina Bulatovic, Milos Gasic, Bojan Jaksic, Milan Stojanovic, Dusica Miljkovic Jaksic, Arijeta Kostic, Roksanda Krivcevic Nikolcevic, Aleksandra Balovic, Filip Petrović

**Affiliations:** 1 Faculty of Medicine in Priština, University of Priština Temporarily Settled in Kosovska Mitrovica, 38220 Kosovska Mitrovica, Serbia; simon.nikolic@med.pr.ac.rs (S.N.); drzelek@gmail.com (Z.E.); jelena_km@hotmail.com (J.A.P.); aleksandra.ilic@med.pr.ac.rs (A.I.); kristinajakovljevic@gmail.com (K.B.); gasic.milos@yahoo.com (M.G.); roksanda.krivcevic@gmail.com (R.K.N.); balovicaleksandra@gmail.com (A.B.); 2 Clinical Hospital Center Priština, 38205 Gračanica, Serbia; arijeta_pr@hotmail.com; 3 Clinical Hospital Center Kosovska Mitrovica, 38220 Kosovska Mitrovica, Serbia; bojanstjaksic@gmail.com (B.J.); dusicamkv@gmail.com (D.M.J.); 4 Radiology Center, Medical Faculty, University Clinical Center Nis and University of Nis, 18000 Niš, Serbia; milance8412@gmail.com (M.S.); fpetrovic91@gmail.com (F.P.)

**Keywords:** COVID-19, chest CT severity score, disease severity, outcome

## Abstract

The aim of this study is to examine the prognostic role of initial chest computed tomography severity score index (CTSS) and its association with demographic, socio-epidemiological, and clinical parameters in COVID-19 hospitalized patients. A retrospective study included patients who were hospitalized in the COVID Hospital of the Clinical Hospital Center Kosovska Mitrovica from July 2020 to March 2022. We compared patient characteristics and outcome of their hospital stay with values of CT severity score (mild, moderate, and severe form of the disease). Patients with severe disease were statistically significantly older, they treated more days, and they presented statistically significant highest mortality rate compared to mild and moderate forms. Smokers and obese were significantly more frequent among patients with higher CT, while vaccinated patients were more common among those with a mild form. Biochemical parameters at admission also showed statistical significance between the examined groups. We can conclude that by employing the initial CT severity score as the strongest predictor of mortality, it is possible to predict the outcome in hospitalized patients. A comprehensive examination of the patient upon admission, including determining the extent of inflammatory changes in the lungs using computed tomography, the levels of oxygen saturation, and other laboratory parameters, can assist doctors in making an adequate clinical evaluation and apply appropriate therapeutic protocols in the treatment of COVID-19.

## 1. Introduction

COVID-19 (coronavirus disease) is an acute infectious disease caused by a new strain of coronavirus called severe acute respiratory syndrome coronavirus-2 (SARS-CoV-2). The disease spread rapidly and reached pandemic proportions, which significantly impacted healthcare systems and socio-economic conditions worldwide [1,2,3]. In the Republic of Serbia, the first case of COVID-19 was confirmed on 6 March2020. By May 2024, a total of 2,615,127 cases were recorded, out of which 18,057 resulted in death [2,4]. Due to the high transmissibility of SARS-CoV-2 and the rapid spread of the infection, early and accurate diagnosis is vital for timely and effective treatment of patients. Diagnosis is based on clinical features, microbiological and serological methods, laboratory analyses, and radiological diagnostics.

The clinical features of COVID-19 are nonspecific and can vary from asymptomatic cases to findings of atypical pneumonia, respiratory failure, and death, or a several-month recovery. The most common symptoms are as follows: fever, dry cough, fatigue, muscle and joint pain, shortness of breath (dyspnea), and headache. Runny nose, sore throat, anosmia, and gastrointestinal symptoms occur less frequently [5,6]. Blood analyses most often detect lymphopenia, while the total leukocyte count may vary. Numerous studies mention significantly higher leukocyte levels coupled with more prominent lymphopenia in cases with fatal outcomes. Thrombocytopenia is also common, along with increased levels of D-dimer and inflammatory markers such as C-reactive protein (CRP), erythrocyte sedimentation rate (ESR), interleukin-6 (IL-6), and procalcitonin. Changes in biochemical parameters have also been observed, including increased levels of enzymes such as lactate dehydrogenase (LDH), aspartate-aminotransferase (AST), alanine-aminotransferase (ALT), and creatine kinase (CK), among others, often accompanied by hypoalbuminemia and hypoproteinemia [6,7,8,9,10,11,12,13]. In patients with COVID-19, the severity of hypoxemia is independently associated with hospital mortality and can be an important predictor of risk for admission to the intensive care unit, even without signs of respiratory distress or dyspnea [6,14].

Research has shown that numerous risk factors influence the clinical course of the disease. Older age has been identified as a factor associated with poor prognosis and mortality [15,16]. Several studies have demonstrated that severe forms of the disease occur in males and in individuals with comorbidities such as hypertension, diabetes mellitus, and chronic kidney failure. Besides this, a significant number of obese patients who required invasive mechanical ventilation have been observed [17,18]. In addition to the listed factors, viral load, the onset of treatment, socio-economic status, lifestyle, geographical differences, ethnicity, and the quality of healthcare also influence individual disease outcomes [13]. Smoking has been shown to be associated with the development of more severe forms of COVID-19 and fatal outcomes [19]. Numerous studies point to the effectiveness of vaccines in preventing COVID-19 or in reducing severe forms of the disease and complications compared to the unvaccinated [20,21,22].

The method for detecting sequences of the viral genome using a nasopharyngeal swab, which represents the gold standard in diagnosing SARS-CoV-2, is the RT-PCR test (reverse-transcriptase polymerase chain reaction). Although this test is highly specific, its sensitivity is lower, which can be attributed to the insufficient quantity of virus in the sample at a given time, as well as technical errors during swab collection and sample processing [23,24,25].

Radiological imaging methods, primarily computed tomography (CT) of the chest, play an essential role in diagnosing COVID-19, planning appropriate treatment, and assessing the response to the administered therapy [26,27]. CT scores or indices have been proposed as useful tools for evaluating the extent of lung changes, which can help predict disease outcomes. One of the most commonly used models is the CT Severity Score Index (CTSS), as proposed by Pan et al. [28]. According to this model, the percentage of parenchyma involvement by inflammatory changes is determined in each lung lobe, which is represented by a scoring system: if there are no changes, the score is 0; when lung parenchyma involvement is less than 5%, the score is 1; when involvement is 5–25%, the score is 2; for 25–50% involvement the score is 3; for 50–75% involvement the score is 4; and a score of 5 is assigned for 75% involvement. The scores of all lobes are summed, with a maximum score of 25. When the total score is less than 7, the disease is considered to be mild, moderate when it is 8–17, and severe when the score is 18–25 [3,28,29].

The aim of our study is to examine the prognostic role of CT scoring of disease severity at hospital admission and its association with demographic, socio-epidemiological, and initial clinical parameters in patients with COVID-19.

## 2. Materials and Methods

### 2.1. Ethical Approval

This research was approved by the Ethics Committee of the Faculty of Medicine in Pristina, University of Pristina Temporarily Settled in Kosovska Mitrovica (decision number 09-1231,13 June 2023) and the Ethics Committee of the Clinical Hospital Center Kosovska Mitrovica, Republic of Serbia (decision number 786, 28 February 2022).

### 2.2. Data Collection

A retrospective study included 176 patients with confirmed SARS-CoV-2 virus infection who were hospitalized in the COVID Hospital of the Clinical Hospital Center Kosovska Mitrovica from July 2020 to March 2022 and were treated according to the valid National Protocol of the Republic of Serbia for the treatment of COVID-19 infection. All participants were referred to the Department of Radiology and Ultrasound Diagnostics for a chest CT scan upon admission. Individuals under the age of 18, pregnant women, PCR-negative patients, and those with incomplete data were excluded from this study.

Health, demographic, socio-epidemiological data, and the outcomes of hospital treatment were obtained from the hospital information system. The COVID-19 diagnosis was confirmed by a positive PCR test (Novel Coronavirus (2019-nCoV) Nucleic Acid Diagnostic Kit (PCR-Fluorescence Probing), Sansure Biotech, Changsha, Hunan Province, People’s Republic of China, which detected nucleic acid from a nasopharyngeal swab sample. Blood tests obtained using standard laboratory methods upon hospital admission were complete blood analysis, C-reactive protein (CRP), D-dimer, fibrinogen, aspartate aminotransferase (AST), alanine aminotransferase (ALT), lactate dehydrogenase (LDH), albumin, total proteins, glucose, creatinine, and urea. Additionally, oxygen saturation and body temperature were measured.

Chest CT scans were performed on a Hitachi Eclos 16 CT scanner according to a standard protocol, except in cases of suspected pulmonary thromboembolism, when postcontrast scanning was also conducted. The region covered by the scan included the area from the upper thoracic aperture to the costophrenic angles. The patient was scanned in a supine position with raised arms, using a breath-holding technique at the end of inspiration. All CT scans were processed on diagnostic workstations and interpreted by two experienced radiologists. By determining the disease severity score (CT Severity Score Index—CTSS), the percentage of lung involvement with pneumonia was established. Based on the obtained values, the form of the disease (mild, moderate, and severe) was assessed, and their association with the clinical, demographic, and socio-epidemiological characteristics of the participants was examined. By monitoring disease outcomes, the prognostic role of the initial chest CT examination of hospitalized patients was evaluated.

### 2.3. Statistical Analysis

For descriptive statistical methods, continuous variables are presented as the arithmetic mean and standard deviation or as median and interquartile range (Q1–Q3), depending on the normality of distribution tested by the Shapiro–Wilk test. The distributions of frequency for categorical variables are shown as absolute and relative numbers. Either the T-test or the Mann–Whitney U test (rank sum test) was used to test the hypothesis about the significance of the difference in means of numerical features. Chi-square and Fisher’s exact probability tests were employed to test the differences in frequencies of categorical variables. The predictive potential of all laboratory parameters and the overall CT score was examined using ROC analysis. The area under the ROC curve and the 95% confidence interval were calculated, as well as the cut-off value with optimal sensitivity and specificity for predicting mortality.

Statistical hypotheses were tested at a statistical significance level (alpha level) of 0.05. All analyses were performed using SPSS Statistics 22 software (SPSS Inc., Chicago, IL, USA).

## 3. Results

This study included 176 patients, aged 18 to 91 years. The average age was 61.5 ± 15.0. Out of the total number of patients, 119 (67.6%) were male and 57 (32.4%) were female.

**Figure 1:**
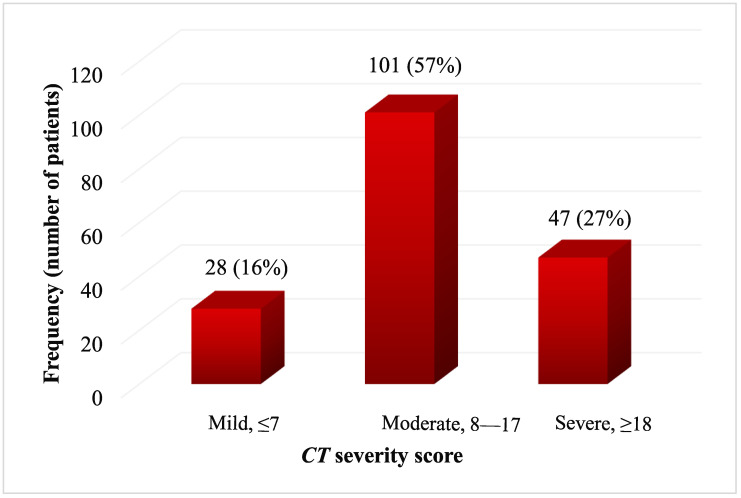
The frequency of patients according to the CT severity score of the disease.

Based on the CT score values, the patients’ clinical appearance caused by COVID-19 infection was divided into three forms, namely, mild (≤7), moderate (8–17), and severe (≥18). The most common form of the disease was moderate, which was observed in more than half of the patients, 101 (57.4%); severe cases were present in 47 (26.7%), while 28 (15.9%) patients had a mild form of the disease (Figure 1).

The form of the disease based on the CT score is a grouping variable used to analyze the association with sociodemographic characteristics, laboratory, clinical parameters, and disease outcomes.

The gender distribution of patients with COVID-19 did not differ statistically significantly with respect to the form of the disease (*p* = 0.473). The age of the patients varied significantly (*p* = 0.001), with those having a mild form being significantly younger compared to the patients with moderate (*p* = 0.001) and severe (*p* = 0.001) forms, while there was no significant age difference between moderate and severe cases (*p* = 0.685). Smokers were significantly more frequent among severe cases (*p* = 0.002). Regarding the prevalence of comorbidities, a statistically significant difference was found for hypertension (*p* < 0.001), diabetes mellitus (*p* = 0.033), and obesity (*p* = 0.018). Hypertension had a statistically significantly lower prevalence in patients with a mild form of the disease (*p* < 0.001). Diabetes mellitus also had a statistically significantly lower prevalence in patients with a mild form of the disease (*p* = 0.017). The prevalence of obesity was statistically significantly higher in severe forms of the disease compared to mild and moderate ones (*p* = 0.005). The vaccination status of patients was significantly associated with the forms of the disease (*p* < 0.001). Vaccinated patients were significantly more frequent among those with a mild form (*p* < 0.001). The prevalence of complete vaccination ranged from 60.0% to 81.0% and did not show a significant association with the form of the disease (*p* = 0.303) (Table 1).

During their hospital stay, hospitalization parameters and symptoms of the disease were monitored. Their distribution, depending on the form of the disease, is presented in Table 2. The duration of symptoms prior to hospital admission did not significantly differ with respect to the form of the disease (*p* = 0.327), while the length of hospital stay was significantly different with respect to the form of the disease (*p* = 0.001). The length of stay for mild cases was 10 (7.2–12.8), for moderate cases was 13 (10–18.5), and for severe cases was 16 (10–25), with a significant difference between mild and moderate forms (*p* = 0.001) as well as between mild and severe forms (*p* = 0.001), while the difference was not significant between moderate and severe cases (*p* = 0.257). None of the patients with mild forms of the disease was transported to the ICU; 17.8% of those with moderate forms were transported, while a significantly higher transport rate of 95.7% (*p* < 0.001) was observed among patients with severe forms. There were no deaths among patients with mild forms, whereas mortality was observed in 13.9% of patients with moderate forms, while the statistically significant highest mortality rate of 89.4% (*p* < 0.001) was noted in patients with severe forms. Symptoms that showed statistically significant differences in frequency concerning the form of the disease were shortness of breath (*p* = 0.001), runny nose (*p* = 0.019), gastrointestinal issues (*p* = 0.021), myalgia, and arthralgia (*p* = 0.042). Among mild cases, the significantly least frequent symptoms were shortness of breath (*p* < 0.001) and gastrointestinal symptoms (*p* = 0.012), each at 10.7%, while the most common symptom in this group was a runny nose, also at 10.7% (*p* = 0.006). Myalgia and arthralgia were significantly the least prevalent among patients with moderate forms, at 21.8% (*p* = 0.015). Body temperature did not significantly differ with reference to the form of the disease (*p* = 0.072).

The laboratory parameters for COVID-19 patients at admission that showed significant differences with reference to the severity of the disease are as follows: SPO_2_ (*p* < 0.001), neutrophils (*p* < 0.001), lymphocytes (*p* < 0.001), CRP (*p* < 0.001), D-dimer (*p* < 0.001), AST (*p* < 0.001), ALT (*p* = 0.008), LDH (*p* < 0.001), albumins (*p* < 0.001), total proteins (*p* < 0.001), glucose (*p* = 0.001), and urea (*p* = 0.002), as shown in Table 3.

The levels of SPO_2_ were statistically significantly higher in the mild form compared to the moderate (*p* < 0.001) and severe forms of the disease (*p* < 0.001). Patients with moderate forms had significantly higher levels compared to those with severe forms (*p* < 0.001). Neutrophil levels were statistically significantly higher in moderate forms compared to mild (*p* = 0.002), in severe forms compared to mild (*p* < 0.001), and in severe forms compared to moderate forms (*p* = 0.035). Lymphocyte levels were statistically significantly higher in mild forms compared to moderate (*p* = 0.002), in mild forms compared to severe (*p* < 0.001), and in moderate forms compared to severe forms (*p* = 0.005). C-reactive protein levels were statistically significantly higher in moderate compared to mild forms (*p* < 0.001), in severe forms compared to mild (*p* < 0.001), and in severe forms compared to moderate ones (*p* = 0.047). D-dimer levels were statistically significantly higher in moderate forms compared to mild (*p* < 0.001), in severe forms compared to mild (*p* < 0.001), and in severe forms compared to moderate ones (*p* = 0.026). AST levels were statistically significantly higher in severe forms compared to mild (*p* < 0.001) and moderate (*p* = 0.001), with no significant difference between mild and moderate forms (*p* = 0.086). ALT levels were statistically significantly higher in severe forms compared to mild (*p* = 0.028) and moderate (*p* = 0.003), with no significant difference between mild and moderate forms (*p* = 0.817). LDH levels were statistically significantly higher in severe forms compared to mild (*p* < 0.001) and moderate (*p* = 0.002), with no significant difference between mild and moderate forms (*p* = 0.079). Albumin levels were statistically significantly higher in mild forms compared to moderate (*p* < 0.001) and severe forms (*p* < 0.001). Albumin levels were also significantly higher in moderate compared to severe forms (*p* = 0.001). Protein levels were statistically significantly higher in mild forms compared to moderate (*p* = 0.003) and severe forms (*p* < 0.001). Protein levels were also significantly higher in moderate compared to severe forms (*p* < 0.001). Glucose levels were significantly lower in mild compared to moderate (*p* = 0.004) and severe forms (*p* < 0.001). There was no significant difference in glucose levels between moderate and severe forms (*p* = 0.115). Urea levels were significantly lower in mild compared to moderate (*p* = 0.004) and severe forms (*p* < 0.001). There was no significant difference in urea levels between moderate and severe forms (*p* = 0.310) (Table 3).

### ROC Analysis for Predicting Mortality

The values of the CT score and other observed laboratory parameters were analyzed with a view to predicting mortality through ROC (receiver operating characteristic) analysis. Thirteen significant factors were identified as predictors of fatal outcomes in patients with COVID-19, among which SPO_2_ was the most reliable predictor (Table 4).

**Figure 2:**
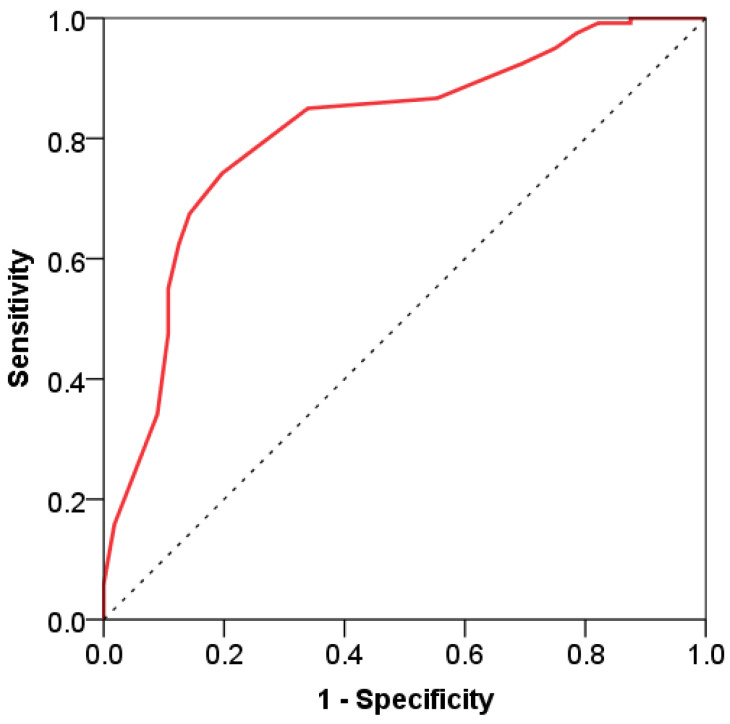
ROC curve of SPO_2_ in predicting mortality in patients with COVID-19.

SPO_2_ is a statistically significant predictor with very good discriminative potential regarding mortality, with an AUC ROC of 0.811 (95% confidence interval 0.745–0.866), *p* < 0.001. The sensitivity value is 80.4%, and the specificity value is 74.2%. SPO_2_ levels below the cut-off value (90.0%) are highly reliable predictors of mortality in patients with COVID-19 (Figure 2).

**Figure 3:**
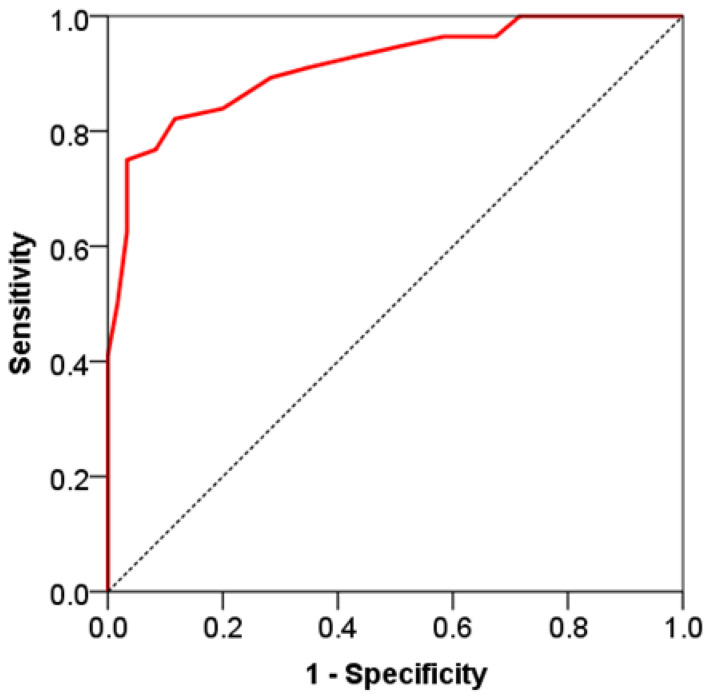
ROC curve of the total CT severity score in the prediction of mortality in patients with COVID-19.

The potential for predicting mortality was also assessed for the total CT score, which showed excellent capacity for discrimination between patients with fatal outcomes and those who survived. AUC ROC for the total CT score is 0.914 (95% confidence interval 0.863–0.951), *p* < 0.001. The sensitivity value is 75.0%, and the specificity value is 96.7%. Total CT score values above the cut-off value (17.0) are an excellent discriminatory criterion for fatal outcome in patients with COVID-19 (Figure 3).

## 4. Discussion

Due to its unpredictable course, numerous studies have attempted to define the pathophysiological mechanisms and other factors affecting the dynamics of COVID-19 development. In this study, we attempted to present an approximate distribution of the characteristics of the affected patients in our region. Our goal was to investigate the connection between the CT score of disease severity and various clinical, demographic, and socio-epidemiological characteristics, as well as the outcomes of hospital treatment.

According to WHO recommendations [30], imaging radiological methods are used as part of the diagnostic evaluation of patients when the RT-PCR test is unavailable or negative but there is clinical suspicion of COVID-19, along with clinical and laboratory parameters in patient triage and in hospitalized patients for assessing therapeutic effect and disease outcomes. The use of chest CT and the determination of the severity score (CTSS) play an important role in the initial assessment of patients with COVID-19 and are positively correlated with inflammatory markers, which can aid in effectively predicting disease severity and the need for admission to intensive care units [31].

Numerous studies have shown that the disease occurs more frequently in older males. Comorbidities such as hypertension, cardiovascular diseases, obesity, diabetes mellitus, and other associated conditions increase the organism’s susceptibility and contribute to the development of more severe forms of COVID-19 [3,5,6,18,32]. Similar results were obtained in our study, where it was observed that older individuals with comorbidities developed a more severe form of the disease. The absence of a statistically significant difference in gender with regard to the disease form is similar to the results of Zhang et al. [12], which can be explained by their differing frequencies, age, associated diseases, and habits, as well as the limited number of patients included in this study.

One of the factors influencing the course of COVID-19 is lifestyle habits, specifically cigarette smoking. A potential explanation is that exposure to cigarette smoke increases both the expression of ACE2 receptors in the respiratory tract of the affected individual and the likelihood of developing a more severe clinical picture [33]. Many studies indicate that a positive smoking status is associated with a more severe form of the disease in intensive care unit patients and mortality [19,34,35,36], which is consistent with our results.

The vaccination status of patients is significantly associated with the form of COVID-19. In our study, there was a significantly higher number of vaccinated patients among those with a mild clinical picture and a CT score of ≤7. This is consistent with other research results pointing to the effectiveness of vaccines in preventing severe disease forms and mortality in affected patients over a limited time period [19,36,37,38].

The average incubation period for COVID-19 is five days, ranging from 0 to 24 days [32,33]. The average duration of symptoms in our patients before hospital admission was 9 days, ranging from 1 to 30 days. In contrast to the results of Stojanović M. et al. [39], the duration of symptoms in our study did not influence the form of the disease. Such discrepancies in relation to the literature data may result from not detecting initial symptoms, unclear reporting of data by patients, or the fact that some patients were treated at home.

Unlike the onset of the disease and the duration of symptoms prior to hospital admission, our study showed that the length of hospital stay was significantly different with reference to the CT score of disease severity, with the longest stays among more severely affected patients. Additionally, an increase in the CT score was associated with a significantly higher number of patients requiring admission to intensive care units, while the statistically significant highest mortality was among patients with a severe form of the disease. These results are consistent with numerous data from the literature [3,29,39,40].

Regarding symptoms, statistically significant differences with reference to the form of the disease in our patients were observed in shortness of breath (dyspnea), runny nose, gastrointestinal symptoms, myalgia, and arthralgia. Shortness of breath occurred significantly more frequently in severe cases and may serve as a warning symptom of a serious condition in patients, indicating a need for admission to intensive care units [41]. In our study, body temperature did not show a statistically significant difference with regard to the form of the disease, which is consistent with the results of Pereto Silva et al. [42].

Oxygen saturation upon admission represents the best predictor of mortality in younger adults [43]. In our study, SPO_2_ levels were significantly lower in patients with CT scores ≥ 18. Laboratory parameters associated with an increased risk of developing severe clinical pictures include neutrophilia, lymphopenia, and increased levels of ALT, AST, LDH, CRP, and ferritin [12,13,17,44], which are consistent with our results. We can assume that lymphopenia results from the virus binding to ACE2 receptors on lymphocytes, making them a target. This leads to damaging the immune system of the patient and an exaggerated systemic immune response, which is accompanied by an increased number of neutrophils. Moreover, neutrophils are thought to be associated with the development of ARDS and can induce thrombosis in patients with COVID-19 [45,46]. Increased levels of LDH, AST, and ALT may be attributed to greater cytotoxic damage in clinically severe cases. Our results are consistent with the research of other authors, which show that high levels of CRP and hypoalbuminemia are significant for the occurrence of severe forms of the disease, indicating the development of cytokine storms [9,43]. Coagulation disorder is a well-known systemic effect of COVID-19 that can occur due to direct or indirect viral impact on the endothelium or as a result of immunothrombosis [7,47]. The level of D-dimer upon admission correlates with disease severity and is a reliable prognostic marker for hospital mortality in COVID-19 patients, either alone [48,49] or in combination with urea levels [50], which is similar to our results. However, in contrast, Bruggeman et al. [51] have said that there was no significant association observed between the CTSS and the occurrence of pulmonary embolism, although its value was higher in patients who required ICU admission. The authors believe that pulmonary embolism is the result of immunothrombosis rather than being a bystander of severe pulmonary inflammation. Hyperglycemia, along with older age of the patient, clinical indicators of systemic inflammatory response, and multiorgan failure, may be significant in predicting disease progression and mortality [52], which was also observed in our study.

Due to all of the above, it is essential to quickly and accurately determine means for identifying patients at the highest risk of death. Numerous risk stratification systems are in use for predicting fatal outcomes in patients with COVID-19 [53]. In our study, the values of the CT severity score and laboratory parameters upon admission were analyzed with the aim of predicting mortality through ROC analysis. Out of all laboratory parameters, the SPO_2_ level was estimated to be the predictor with the highest reliability in our research, which is consistent with many previous studies [6,43,54,55]. These results may suggest that a large percentage of our patients were hospitalized too late after developing significant hypoxemia, which resulted in higher mortality rates.

The potential for predicting fatal outcomes was also assessed for the overall CT disease severity score, where scores above the cut-off value (17.0) were identified as an excellent discriminative criterion for fatal outcomes in patients with COVID-19. Our results are similar to those of Francone M. et al. [56], where scores ≥ 18 were predictors of mortality, as well as to the results in many other studies [57,58]. In contrast to these results, Nokiani et al. [59] considered that CTSS is an excellent tool in triage and prognostication in patients with COVID-19 ≥ 65 years old but is of limited value in younger patients, which could be explained by the different patient sample and different CT scoring system compared to our study.

Compared to SPO_2_, the CT disease severity score has a greater prognostic value in assessing outcomes for hospitalized patients.

## 5. Conclusions

Based on our research, we can conclude that by employing the initial CT severity score as the strongest predictor of mortality, it is possible to predict the outcomes of hospital treatment of patients with COVID-19. A comprehensive examination of the patient upon admission, including determining the extent of inflammatory changes in the lungs using computed tomography, the levels of oxygen saturation, and other laboratory parameters, can assist doctors in making an adequate clinical evaluation and, consequently, in applying appropriate therapeutic protocols in the treatment of COVID-19.

Our study has several limitations. First, it is a retrospective study. Additionally, the majority of patients included in our research had a moderate form of the disease (CT score 8–11) compared to mild and severe forms. There was a low percentage of vaccinated patients in our sample. There was no opportunity to follow up on the condition of the survivors after hospital discharge. Finally, this is a single-center study and, as such, is limited to a specific geographic region, which could reduce the generalization of our findings.

## Figures and Tables

**Table 1:** Main demographic characteristics, habits, and comorbidities of patients according to the severity of the disease.

	*CT* Severity Score			*p*
	Mild	Moderate	Severe	
Total Number (%)	28 (16.0%)	101 (57.0%)	47 (27.0%)	
Gender				
Male	19 (67.9)	65 (64.4)	35 (74.5)	0.473
Female	9 (32.1)	36 (35.6)	12 (25.5)	
Age	47.5 (34.2–64.5)	64.0 (55.0–74.0)	67.0 (57.0–73.0)	0.001 *
Smoking history				
No	18 (64.3)	54 (53.5)	10 (21.3)	
Yes	4 (14.3)	25 (24.8)	22 (46.8)	0.001 *
Former smoker	6 (21.4)	22 (21.8)	15 (31.9)	
Comorbidities				
Arterial hypertension	9 (32.1)	64 (63.4)	40 (85.1)	<0.001 *
Cardiovascular diseases	6 (21.4)	22 (21.8)	15 (31.9)	0.378
Diabetes mellitus	2 (7.1)	26 (25.7)	16 (34.0)	0.033 *
COPD	0 (0.0)	1 (1.0)	3 (6.4)	0.083
Neurological diseases	1 (3.6)	6 (5.9)	3 (6.4)	0.866
Psychiatric diseases	0 (0.0)	4 (4.0)	2 (4.3)	0.553
Malignant conditions	1 (3.6)	9 (8.9)	2 (4.3)	0.439
Obesity	6 (21.4)	26 (25.7)	22 (46.8)	0.018 *
Vaccination status				
Unvaccinated	7 (25.0)	76 (75.2)	40 (85.1)	
Vaccinated	21 (75.0)	25 (24.8)	7 (14.9)	<0.001 *
Vaccination				
Incomplete	4 (19.0)	10 (40.0)	2 (28.6)	0.303
Complete	17 (81.0)	15 (60.0)	5 (71.4)	

Numerical data are presented as median (Q1-Q1), and categorical data are presented as number of patients and percentage, n (%). * Statistically significant value.

**Table 2:** Clinical characteristics on admission according to the severity of the disease.

	*CT* Severity Score			*p*
	Mild	Moderate	Severe	
Total Number (%)	28 (16.0%)	101 (57.0%)	47 (27.0%)	
Duration of symptoms before admission (days)	7 (2.2–10)	8 (5–12)	7 (5–14)	0.327
Duration of hospital stay(days)	10 (7.2–12.8)	13 (10–18.5)	16 (10–25)	0.001 *
ICU admission				
No	28 (100.0)	83 (82.2)	2 (41.3)	
Yes	0 (0.0)	18 (17.8)	45 (95.7)	<0.001 *
Disease outcome				
Discharged	28 (100.0)	87 (86.1)	5 (10.6)	<0.001 *
Died	0 (0.0)	14 (13.9)	42 (89.4)	<0.001 *
Symptoms				
Fever	27 (96.4)	94 (93.1)	43 (91.5)	0.712
Cough	14 (50.0)	57 (56.4)	31 (66.0)	0.357
Dyspnea	3 (10.7)	42 (41.6)	25 (53.2)	0.001 *
Sore throat	1 (3.6)	7 (6.9)	3 (6.4)	0.809
Chest tightness	3 (10.7)	11 (10.9)	11 (23.4)	0.108
Runny nose	3 (10.7)	2 (2.0)	0 (0.0)	0.019 *
Fatigue	20 (71.4)	75 (74.3)	38 (80.9)	0.587
Headache	8 (28.6)	13 (12.9)	9 (19.1)	0.134
Gastrointestinal symptoms	3 (10.7)	38 (37.6)	13 (27.7)	0.021 *
Anosmia	2 (7.1)	9 (8.9)	2 (4.3)	0.601
Myalgia/arthralgia	12 (42.9)	22 (21.8)	17 (36.2)	0.042 *
Body temperature (°C)	37.8 (37.4–38.0)	37.8 (37.5–38.4)	38.0 (37.5–39.0)	0.072

Numerical data are presented as median (Q1-Q1), and categorical data are presented as number of patients and percentage, n (%). * Statistically significant value.

**Table 3:** Laboratory parameters on admission.

Parameters	*CT* Severity Score			*p*
	Mild	Moderate	Severe	
Total Number (%)	28 (16.0%)	101 (57.0%)	47 (27.0%)	
SPO_2_ (%)	96.5 (95.0–98.0)	93.0 (90.0–96.0)	88.0 (85.0–90.0)	<0.001 *
Leukocytes (×10^9^/L)	6.9 (4.9–10.9)	7.4 (5.5–12.0)	9.2 (7.0–13.0)	0.166
Neutrophils (%)	0.7 (0.6–0.8)	0.8 (0.8–0.9)	0.9 (0.8–0.9)	<0.001 *
Lymphocytes (%)	0.2 (0.1–0.3)	0.1 (0.1–0.2)	0.05 (0.07–0.14)	<0.001 *
Erythrocytes (×10^12^/L)	4.4 (4.1–4.7)	4.5 (4.0–4.8)	4.5 (4.0–4.9)	0.574
Hemoglobin (g/L)	130.5 (122.2–139.0)	132.0 (119.0–141.5)	130.0 (120.0–144.0)	0.815
Platelets 10^9^/L	221.0 (145.5–275.8)	190.0 (140.5–286.5)	206.0 (144.0–279.0)	0.776
CRP (IU/mL)	20.5 (8.0–65.8)	84.0 (38.5–139.0)	108.0 (65.0–163.0)	<0.001 *
D-dimer (ng/mL)	404.0 (221.5–630.5)	1000.0 (443.0–2410.0)	1870.0 (1000.0–2300.0)	<0.001 *
Fibrinogen (g/L)	5.6 (4.6–6.5)	5.9 (5.0–7.0)	6.2 (4.6–7.1)	0.517
AST (U/L)	29.6 (22.2–38.2)	37.0 (27.0–50.0)	54.0 (32.0–75.0)	<0.001 *
ALT (U/L)	40.5 (24.0–49.0)	34.0 (25.0–46.5)	50.0 (32.0–76.0)	0.008 *
LDH (U/L)	413.0 (307.5–501.8)	468.0 (371.5–589.0)	566.0 (444.0–762.0)	<0.001 *
Albumin (g/L)	42.0 (37.2–44.0)	35.0 (31.0–39.5)	33.0 (29.0–35.0)	<0.001 *
Total proteins (g/L)	71.0 (64.0–75.0)	67.0 (60.5–69.0)	62.0 (56.0–66.0)	<0.001 *
Glucose (mmol/L)	6.0 (5.4–7.0)	7.5 (6.0–10.0)	7.9 (6.1–13.9)	0.001 *
Creatinine (mmol/L)	81.0 (76.2–94.5)	84.0 (68.5–107.5)	82.0 (69.0–105.0)	0.975
Urea (mmol/L)	5.2 (4.0–5.8)	6.9 (4.8–9.4)	7.1 (5.4–11.0)	0.002 *

Numerical data are presented as median (Q1-Q1), and categorical data are presented as number of patients and percentage, n (%). * Statistically significant value. Abbreviations: SPO_2_: oxygen saturation; CRP: C reactive protein; AST: aspartate aminotransferase; ALT: alanine aminotransferase; LDH: lactate dehydrogenase.

**Table 4:** Values of laboratory parameters in the prediction of fatal outcome in patients with COVID-19.

Parameters	AUC ROC	*p* Value	95% Confidence Interval		Cutt-Of Value	Sens. (%)	Spec. (%)
			Lower Limit	Upper Limit			
Duration of symptoms before admission (days)	0.592	0.059	0.515	0.665	>18.0	41.1	80.8
Duration of hospital stay (days)	0.529	0.522	0.453	0.605	>2.0	98.2	12.5
Body temperature (°C)	0.601	**0.029**	0.525	0.674	>37.9	64.3	54.2
SPO_2_	0.811	**<0.001**	0.745	0.866	<90.0	80.4	74.2
Leukocytes (×10^9^/L)	0.613	**0.014**	0.537	0.685	>7.1	71.4	50.0
Neutrophils (%)	0.683	**<0.001**	0.609	0.751	>0.9	33.9	92.5
Lymphocytes (%)	0.682	**<0.001**	0.607	0.750	<0.08	58.9	74.2
Erythrocytes (×10^12^/L)	0.551	0.292	0.474	0.626	>4.8	35.7	82.5
Hemoglobin (g/L)	0.533	0.506	0.456	0.608	>139.0	35.7	76.7
Platelets (10^9^/L)	0.506	0.900	0.430	0.582	<175.0	44.6	65.0
CRP (IU/mL)	0.613	**0.009**	0.537	0.686	>57.0	82.1	43.3
D-dimer (ng/mL)	0.649	**<0.001**	0.574	0.719	>770.0	82.1	51.7
Fibrinogen (g/L)	0.562	0.202	0.485	0.636	>6.4	48.2	71.7
AST (U/L)	0.653	**<0.001**	0.578	0.723	>37.0	71.4	59.2
ALT (U/L)	0.612	**0.022**	0.535	0.684	>45.0	50.0	74.2
LDH (U/L)	0.649	**<0.001**	0.573	0.719	>500.0	60.7	63.3
Albumin (g/L)	0.698	**<0.001**	0.624	0.765	<35.0	76.8	57.5
Total proteins (g/L)	0.688	**<0.001**	0.614	0.755	<67.0	78.6	51.7
Glucose (mmol/L)	0.555	0.239	0.478	0.630	>5.8	83.9	27.5
Creatinine (mmol/L)	0.517	0.716	0.440	0.592	>65.2	92.9	65.2
Urea (mmol/L)	0.627	**0.004**	0.551	0.699	>5.7	76.8	46.7

Bolded—statistically significant predictor of fatal outcome (*p*< 0.05), AUC ROC—area under the ROC curve, Sens.—sensitivity, Spec.—specificity, Abbreviations: SPO_2_: oxygen saturation; CRP: C reactive protein; AST: aspartate aminotransferase; ALT: alanine aminotransferase; LDH: lactate dehydrogenase.

## Data Availability

The data presented in this study are available on request from the corresponding author.
